# 

**Published:** 1893-06

**Authors:** 


Whole Number, CCCLXXXI.

New Series.

June, 1893.

VOL. XXXII. No. 11.

Indexed S. G. O. LIBRARY

### Buffalo Medical and Surgical Journal.

Established 1845 by AUSTIN FLINT, M. D.

**Editors:**

THOS. LOTHROP, M. D. WM. WARREN POTTER, M. D.

**Associate Editors:**

WILLIAM C. KRAUSS, M. D.

JAMES WRIGHT PUTNAM, M. D.

JOHN A. MILLER, Ph. D.

DeLANCEY ROCHESTER, M. D.

JOHN PARMENTER, M. D.

SCRIPTA FERUNT ANNOS

European Agency,
TRÜBNER & CO.
57 and 59 Ludgate Hill, London, E. C.

BUFFALO:
1893.

Foreign Subscription, $2.50

SUBSCRIPTION PRICE, $2.00 IF PAID IN ADVANCE; OTHERWISE $2.50 A YEAR.

PUBLISHED MONTHLY.

Entered at the Post-Office at Buffalo, N. Y., as Second-class Mail Matter.

*Times Printing House, Buffalo, N. Y.*

